# Implications of Stabilometric Assessment in Determining Functional Deficits in Patients with Severe Knee Osteoarthritis: Observational Study

**DOI:** 10.3390/jcm13113181

**Published:** 2024-05-29

**Authors:** Marius Neculăeș, Pablo Hernandez-Lucas, Paul Lucaci

**Affiliations:** 1 Faculty of Physical Education and Sport, “Alexandru Ioan Cuza” University of Iași, 3 Toma Cozma Street, 700554 Iasi, Romania; marius.neculaes@uaic.ro (M.N.); paul.lucaci@uaic.ro (P.L.); 2 Faculty of Physiotherapy, University of Vigo, Campus A Xunqueira, 36005 Pontevedra, Spain

**Keywords:** center of gravity, functionally delayed, physiotherapy, posturograph

## Abstract

**Background**: Osteoarthritis is one of the most frequent joint disorders in the world. The specialists in the field strongly support the role of physical exercise as a key component in the holistic management of arthrosis. The aim of the current study was to identify and assess the functional deficit of these patients and to identify means to alleviate it through pre-surgery physiotherapy programs. **Methods**: The study was conducted on two samples of patients: a witness sample, encompassing 126 subjects without pathologies at the level of their lower limbs, and a study sample, formed of 116 subjects diagnosed with severe gonarthrosis with total knee arthroplasty indication. The assessment protocol was accomplished with the GPS 400 stabilometric platform. **Results**: The barycenter differences within the support polygon, recorded for the two samples within sagittal deviation, emphasize that the barycenter shifting mainly towards the healthy lower limb will demand, from the individual, more intense rebalancing postural reactions that will place the center-of-gravity projection in the sagittal plane, closer to the central area of the support polygon. **Conclusions**: In the case of gonarthrosis and other joint disorders, the use of functional testing to assess body weight distribution and center-of-gravity imbalances represents a promising direction in the research on and management of these disorders, providing essential information for functional diagnosing and thus enabling the elaboration and monitoring of individualized functional rehabilitation plans.

## 1. Introduction

Osteoarthritis signifies the breakdown in joint repair due to stresses originating from abnormalities in any joint or periarticular tissue. While cartilage loss is central, osteoarthritis affects the entire joint. Progression rates differ among individuals and within knees over time, with symptoms and signs including pain, stiffness, diminished joint mobility, and muscle weakness. Long-term effects may entail decreased physical activity, deconditioning, disrupted sleep, fatigue, depression, and disability [1]. Pain associated with osteoarthritis typically correlates with physical activity, as in the case of knee osteoarthritis, wherein common activities like stair climbing, rising from a chair, and walking extended distances often trigger discomfort. Morning stiffness typically subsides within half an hour. Patients commonly report a sensation of their knees “giving way”, indicating a symptom of instability [2].

The non-invasive diagnosis of knee osteoarthritis typically relies on physical examination and common imaging techniques such as radiography (X-ray), computed tomography (CT), magnetic resonance imaging (MRI), and ultrasound [3]. Other recent studies have pointed to vibroarthrography as a cheaper, non-invasive, safe method and an important tool in detecting degenerative changes that occur in the knee joints [4].

Osteoarthritis is one of the most frequent joint disorders worldwide, with some studies emphasizing that one-third of all adults show radiological signs specific to arthrosis [5]. Globally, it is estimated that there are 240 million people featuring symptomatology and the limitation of active movements [6], with more than 27 million people affected by these symptoms in the U.S. [7].

Over the last two decades, it has become evident that the development of OA is triggered by multiple factors, with more recent research also pointing to the role of gut microbiota in the onset of various metabolic and inflammatory conditions [8].

Current studies emphasize that knee osteoarthritis also has an increasing incidence within the younger population, with overweight conditions or former traumatic disorders representing significant factors that contribute to the occurrence and development of this pathology [9]. Furthermore, certain high-performance sport activities can also favor the onset of gonarthrosis at young ages [10]. Recent prospective studies have indicated obesity as a key risk factor for developing knee osteoarthritis, with this connection being strongly supported by biomechanical and metabolic factors [11].

The literature in the field emphasizes the efficiency of physical exercise in improving the functionality of the arthritic knee joint [12], with the data obtained from a sample of 9825 patients diagnosed with hip and knee arthrosis proving the efficiency of physiotherapeutic procedures applied over 6 weeks [13,14]. The most recommended types of exercise for patients with osteoarthritis include aerobics, muscle-strengthening movements [15], and tai chi exercise for releasing pain and improving mobility [16]. Water exercises are also thought to be beneficial and are often recommended for people with osteoarthritis [17]. 

International treatment guides recommend non-pharmacological treatment strategies such as therapeutic exercise, the patient’s medical education, and weight loss when necessary [13,18,19]. Platelet-rich plasma (PRP) treatment may also expedite and sustain therapeutic benefits as noticeable effects of the procedure emerge within the initial six weeks, with subsequent improvements persisting in a satisfactory manner [20].

Despite being widely used, Non-Steroidal Anti-Inflammatory Drugs are frequently associated with adverse drug reactions. This is partly due to their common co-administration with various other drugs across diverse therapeutic scenarios, leading to potential polypharmic interactions [21]. NSAIDs and knee intra-articular corticosteroid injections are also recommended by other higher-quality guidelines [22]. To minimize potential risks, nowadays, artificial intelligence methods have become paramount in classification and regression tasks, being extensively employed across various life and industrial domains for their capacity to learn and adapt to complex data patterns [3].

The specialists in the field strongly support the role of physical exercise, along with patient education and weight management, as key components in the holistic management of arthrosis. These non-pharmacological approaches not only improve joint functionality but also contribute to better overall health and quality of life for individuals living with arthrosis.

The functional assessments of the osteoarthritic knee are required to determine joint mobility limitations and muscle strength and decreases in joint stability and in the ability to achieve correct gait from a biomechanical point of view. Studies conducted in order to determine the degree of joint functionality also use assessments such as of pain levels and the occurrence of joint deformity [23,24].

Functional assessments for osteoarthritic knees encompass a comprehensive evaluation of various aspects including joint mobility, muscle strength, stability, gait patterns, pain levels, and joint deformities. These assessments aid in understanding the extent of impairment, guiding treatment decisions and monitoring the progression of osteoarthritis while striving to improve the quality of life for affected individuals.

Stabilometric assessment is performed in order to determine the weight distribution level in the lower limbs, as well as a series of other load and balance parameters, as indicated by studies carried out regarding stabilometric assessment in traumatic pathologies of the knee [25,26], in ORL and in the field of neuro-motor rehabilitation [27,28].

Stabilometric assessment is a valuable tool in clinical settings, offering insights into weight distribution, balance parameters, and postural control. Its applications range from orthopedic evaluations in traumatic knee injuries to aiding in the assessment and rehabilitation of individuals with balance disorders in fields like ORL and neuro-motor rehabilitation. By providing objective data on balance and weight distribution, stabilometric assessments contribute significantly to understanding and addressing various musculoskeletal and neurological conditions impacting an individual’s balance and stability.

In knee gonarthrosis, testing with the help of the force plate is a method used to analyze and quantify joint stability and balance changes. Knee arthrosis may severely affect normal functionality, and stabilometry implies a series of important measurements such as of weight distribution on the lower limbs’ level and changes in pressure and body position in orthostatism.

Utilizing a force plate for stabilometric assessments in knee gonarthrosis has proved to be a valuable clinical approach as it allows for the quantitative analysis of joint stability, weight distribution, pressure changes, and body positioning alterations, providing crucial insights into the functional implications of knee arthrosis. These objective data support clinicians in designing targeted interventions to improve stability and balance in individuals affected by knee osteoarthritis.

Functional testing through stabilometry is part of the complex assessment that includes the patient’s clinical and paraclinical examination, along with other specific tests, to provide the most complete image possible of the knee joint affected by degenerative disorders that may significantly impact the patient’s quality of life [26]. 

The integration of specialized apparatus for functional testing has emerged as a valuable approach in assessing various pathologies, including gonarthrosis, and holds significant promise in developing tailored pre-surgery functional re-education programs.

Particularly noteworthy is the insight provided by body weight distribution on the healthy lower limb, offering crucial details about body stability and postural adjustments in the sagittal plane, especially evident in patients with severe degenerative knee pathologies.

This finding aligns with corroborative research, underlining how lower-limb pathologies can impact weight distribution and barycenter imbalances, possibly indicating degenerative joint lesions. Understanding the nuanced ways in which joint disorders such as gonarthrosis influence weight distribution and balance presents novel avenues for personalized treatment interventions. 

These insights hold potential to guide therapeutic and functional rehabilitation approaches, significantly alleviating functional limitations in affected patients. Functional testing for evaluating body weight distribution and center-of-gravity imbalances in conditions such as gonarthrosis represents a promising frontier in research and management, offering vital insights for functional diagnostics and the development of personalized rehabilitation plans.

The aim of the current study was to identify and assess the functional deficit of these patients and to identify means to alleviate it through the pre-surgery physiotherapy programs.

## 2. Materials and Methods

### 2.1. Study Participants

The study was conducted in accordance with the Declaration of Helsinki and was approved by the Committee on Scientific Research Conduct and Ethics at the Faculty of Physical Education and Sport, “Alexandru Ioan Cuza” University of Iași, Romania. Written informed consent was obtained from all patients for the publication of this document.

The study was conducted on two groups of patients: a control group, encompassing 126 subjects without pathologies at the level of their lower limbs, and a study group, formed of 116 subjects diagnosed with severe gonarthrosis with total knee arthroplasty indication. 

A series of inclusion criteria were established in order to form the groups. For the control group, the criteria were as follows: age between 50 and 70 and absence of lower-limb pathologies, balance disorders, or neurological diseases. For the study group, the criteria were as follows: age between 50 and 70, clinical diagnosis of severe gonarthrosis with total knee arthroplasty indication, absence of balance disorders and of neurological and neuropsychiatric diseases, and absence of other surgeries at lower-limb level (endo-prosthetics of other joints, post-fracture osteo-synthesis, partial amputations).

### 2.2. Study Protocol

The assessment protocol was accomplished with the GPS 400 stabilometric platform, with the help of which the following parameters were analyzed: balance, center of gravity position in the support polygon, and loading degree for each lower limb.

In this research, we performed the assessment of the barycenter position and that of body weight distribution at the level of lower limbs for the 2 studied groups.

The initial and final assessment were carried out by the same physiotherapist, who has had more than 20 years of expertise in the field, at Kinego clinic in Iasi, Romania, in a controlled environment, at 21 °C, between 09:00 and 16:00 h. All participants were required to wear comfortable clothing and sign the informed consent form prior to the assessment, in compliance with the Helsinki guidelines. 

The testing protocol consisted of assessing body weight distribution at the level of lower limbs and barycenter position from orthostatic position, with open eyes, on the stabilometric platform. Each examination was made without shoes on, the assessment time being of 30 s, during which period the subject maintained the vertical position without postural corrections. Two consecutive tests were performed, 5 min apart, with the patient getting off the platform and resting in sitting position between the tests. This aspect was necessary in order to emphasize the loading stereotype specific to each patient. We considered the average values obtained in the 2 tests for each assessed parameter.

### 2.3. Statistical Analysis

Descriptive statistics such as mean and standard deviation were used to summarize the data. The normal distribution of the groups was assessed using the Shapiro–Wilk test, and homogeneity was confirmed using Levene’s test.

Paired-sample *t*-test was used to identify significant differences in the mean values obtained by the 2 groups. The test allowed us to identify the initial load differences between the left lower limb and the right lower limb for the control group and the load difference between the healthy and the affected lower limb for the study group. The differences between the mean values obtained by the two groups were statistically significant if the significance threshold was less than 0.05 (*p* < 0.05).

The Pearson correlation test was used to highlight the existing associations between the evaluated parameters in the control group and the study group. Depending on the value of the correlation coefficient (r), associations can have a high level of correlation (r > 0.75), a good level of correlation (r = 0.5–0.75), and a moderate level of correlation (r < 0.5), if *p* < 0.05. To identify the difference in loading of the lower limbs for the two groups, *t*-test was used. All statistical analyses were performed in Romania using IBM SPSS Statistics 20.

## 3. Results

In Table 1, we present the descriptive statistics of the two groups included in the study.

In our study, we analyzed the values obtained from the barycenter deviation analysis within the support polygon from body weight distribution at lower-limb level and the correlation of these results between the two groups.

The assessment of weight distribution at lower-limb level in the control group emphasized significant differences, from static point of view (*p* = 0.004), between the right-lower-limb loading and left-lower-limb loading (Table 2).

The increased average on the right lower limb, namely 51.57%, suggests a tendency of accentuated support on the dominant lower limb, considering the type of daily activities and dynamic stereotype. 

These differences are specific to muscle asymmetries and cerebral domination, which imply important changes in biomechanics and joint load at lower-limb level.

As far as the body weight distribution at lower-limb level was concerned, in the case of the study group, loading differences were noticed between the healthy and the affected lower limbs, identifying, after statistical interpretation, a significant value (*p* = 0.000) with a weight distribution mean of 62.37% in favor of the healthy lower limb.

Significant changes were identified regarding the support of the two lower limbs, with higher loading on the healthy lower limb (mean 62.37). This represented a way to adapt to the mechanical stress on the affected lower limb (Table 3).

Persistent and high-intensity pain determines, by reflex, the weight transfer on the healthy limb to avoid the mechanical stress that intensifies pain. The healthy-lower-limb loading degree increases according to pain level and structural affectation at the level of the knee with gonarthrosis.

Regarding the lower-limb loading difference and the sagittal deviation in the witness sample, a significant negative correlation could be noticed, provided by the correlation coefficient value r = −0.190 and by the significance threshold, which had a value of 0.033.

The negative correlation between the lower-limb loading degree and the barycenter deviation in the sagittal plane suggests that a small loading difference between the two lower limbs often allows the possibility of increased barycenter deviations in the anterior–posterior plane. This represents a consequence of the subjects’ postural reactions in orthostatic position and possible postural disorders (Table 4).

A moderate positive association was also noticed between the lower-limb loading difference and lateral deviation, emphasized by the correlation coefficient value r = 0.614 and the significance threshold value *p* = 0.000.

The moderate positive correlation between the lower-limb loading difference and the barycenter frontal deviation confirmed the plantar pressure degree and the loading stereotype of subjects from the control group. The moderate loading asymmetries were confirmed by the moderate shifting of the barycenter towards the support leg.

Within the study group, a positive statistical correlation was noticed between the healthy-lower-limb loading and sagittal deviation according to the correlation coefficient r = 0.196 and to the significance threshold *p* = 0.035 

The low value of the correlation coefficient between the healthy-lower-limb loading and anterior–posterior barycenter shift emphasized the fact that the asymmetrical support with predominant loading on the healthy lower limb required a better projection of the center of gravity within the support polygon in the sagittal plane.

In the study group, a better correlation was also identified between the loading difference and the lateral deviation according to the correlation coefficient r = 0.776 and the significance threshold *p* = 0.000 (Table 5).

The barycenter shift in the frontal plane confirms that the existence of important deviations of the center of gravity in this plane is due exclusively to the healthy lower limb (the support one), which presents a good functional retard and is able to provide body support and stability in orthostatic and dynamic positions.

**Figure 1:**
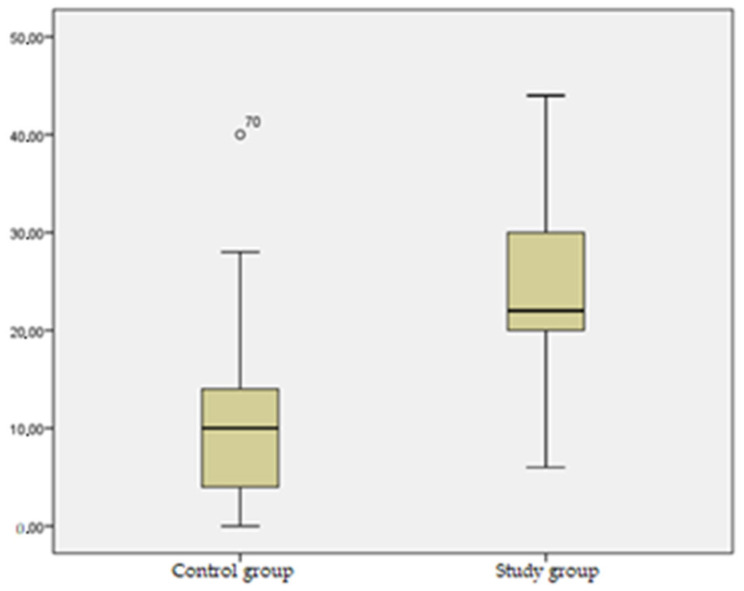
Mean lower-limb loading difference between the control and the study groups.

Regarding the loading difference between the two groups (Table 6, Figure 1), a significant difference was noted, from a statistical point of view (*p* = 0.000), between the left-lower-limb loading and the right-lower-limb loading in the control group (mean 10.42), in comparison with the healthy-lower-limb mean loading and affected-lower-limb mean loading within the study group (mean 24.93), in favor of the healthy lower limb.

The comparative assessment of body weight distribution on the lower limbs between the two groups emphasized the important support asymmetry as a consequence of protecting the knee joint of the affected lower limb, with a significant load increase on the healthy lower limb in patients diagnosed with severe gonarthrosis who had total knee arthroplasty indication.

**Figure 2:**
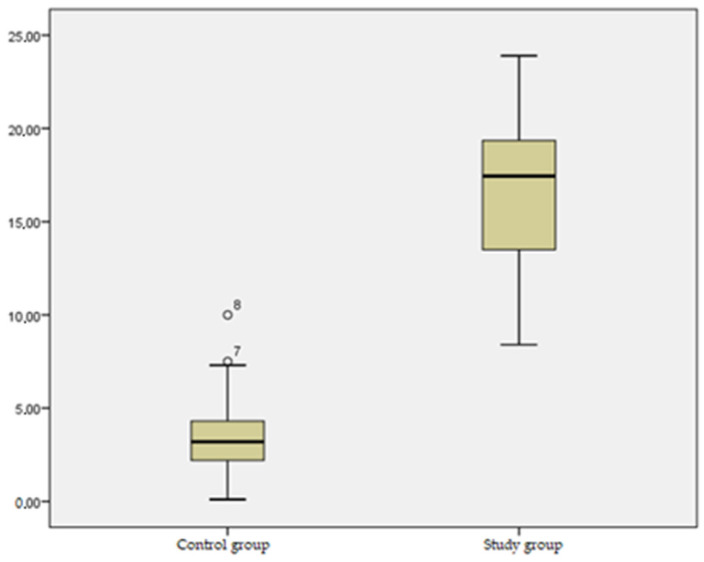
Mean barycenter lateral deviation difference between the two groups.

As shown in Table 7 and Figure 2, a statistically significant deviation (*p* = 0.000) with regard to the mean lateral deviation difference was noted between the two groups, indicating, quite clearly, the far more accentuated lateral deviation tendency within the study group (mean: 16.50) compared to the lateral deviation tendency (mean: 3.36) in the control group.

**Figure 3:**
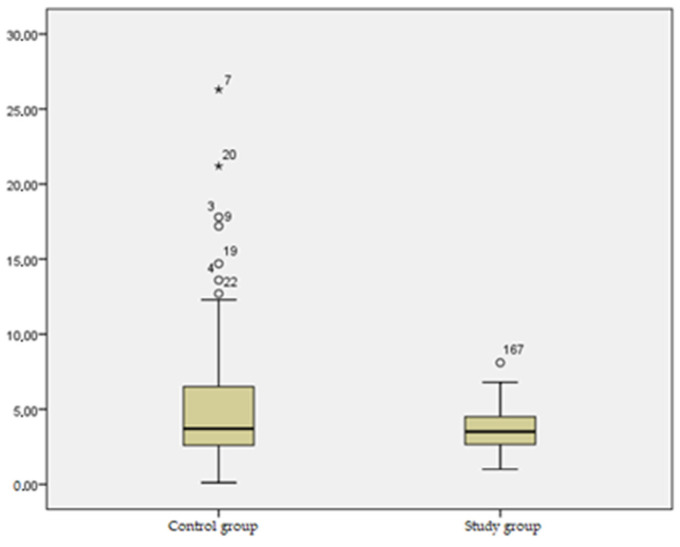
Mean difference in barycenter sagittal deviation between the two groups.

Regarding the mean difference in sagittal deviation between the two groups (Table 8, Figure 3), a statistically significant difference (*p* = 0.000) was noticed, with a decreased deviation tendency in the study group, with a mean of 3.72, compared to the control group, where mean values of 5.20 were identified.

The barycenter differences within the support polygon, recorded for the two groups within sagittal deviation, emphasized that the barycenter shifting mainly towards the healthy lower limb would require more intense rebalancing postural reactions from the subject, and thus, the center of gravity projection would be placed in the sagittal plane, closer to the central area of the support polygon.

## 4. Discussion

To increase the quality of assessments accomplished for various pathologies, including gonarthrosis, functional testing performed in recent years with the help of various devices aimed at measuring different parameters, such as the body weight distribution on the lower limbs and pressure degree on the plantar level, barycenter position within the support polygon, center of gravity velocity, and oscillation amplitude, has known an unprecedented development [29,30].

These parameters are crucial in evaluating functional capabilities, gait patterns, stability, and balance in individuals with various pathologies including gonarthrosis. The use of specialized apparatus to measure these parameters has allowed for more precise and detailed assessments, enabling healthcare professionals to better understand the impact of pathologies on an individual’s movement and functionality.

As indicated in Table 2, the assessment of weight distribution at the level of the lower limbs within the observed cohort highlights substantial disparities, particularly from a static standpoint (*p* = 0.004), between the loading on the right lower limb compared to the left lower limb. The notably higher average loading observed on the right lower limb, accounting for 51.57% on average, indicates a distinct inclination towards increased support provided by the dominant lower limb. This inclination is often influenced by the nature of daily activities and established dynamic movement patterns.

The significant discrepancy in loading between lower limbs suggests potential underlying factors contributing to this asymmetry. Muscle imbalances and the concept of cerebral dominance play pivotal roles in dictating these variations. Muscle imbalances could be attributable to various factors such as habitual movement patterns or occupational activities that exert differential stress on the lower limbs. Furthermore, the notion of cerebral dominance, wherein one hemisphere of the brain exhibits greater influence over motor control and movement, may also contribute to this observed asymmetry. These asymmetries bear implications for biomechanical adjustments and joint loading dynamics within the lower limbs.

This disparity in weight distribution not only reflects mechanical differences between lower limbs but also implies potential alterations in gait patterns, functional movement, and joint loading dynamics. Understanding and addressing these asymmetries are essential in devising tailored rehabilitation or conditioning programs aimed at mitigating potential injury risks, optimizing movement patterns, and restoring more balanced lower-limb functionality.

The results obtained within this study regarding body weight distribution at the lower-limb level in the case of healthy individuals are significant for the dominant lower limb, as indicated by De Blasiis P and co.’s study (2023), in which the existing associations were presented between body stability and weight distribution at the lower-limb level, emphasizing the postural adjustments that may occur in response to various stimuli or according to cerebral domination [31].

In examining the body weight distribution across the lower limbs within the study group (Table 3), discernible differences in loading between the healthy and the affected lower limbs are evident. Statistical analysis indicates a noteworthy and significant discrepancy (*p* = 0.000), with an average weight distribution of 62.37%, favoring the healthy lower limb over the affected one.

These findings underscore substantial alterations in support between the two lower limbs, emphasizing a higher load on the healthy lower limb, with an average of 62.37%. This asymmetrical loading pattern serves as an adaptive response to manage the mechanical stress experienced by the affected lower limb, commonly afflicted with gonarthrosis.

The persistence and intensity of pain associated with the affected lower limb play a critical role in this weight distribution asymmetry. High-intensity pain triggers a reflexive mechanism prompting a transfer of weight onto the healthier limb. This shift in loading is an adaptive strategy aimed at reducing mechanical stress on the affected limb, subsequently alleviating the intensity of pain experienced. Notably, the degree of loading on the healthy lower limb exhibits a direct correlation with both pain intensity levels and the structural impact at the knee affected by gonarthrosis.

This observed weight-distribution imbalance is indicative of the intricate interplay between pain perception, joint pathology, and adaptive mechanisms adopted by the body. It elucidates the body’s innate tendency to redistribute weight asymmetrically as a protective response against heightened pain and structural impairment, shedding light on the complexities of biomechanical adaptations in individuals with gonarthrosis. Understanding these adaptive mechanisms is crucial for tailored interventions aimed at managing pain, optimizing function, and restoring a more balanced weight distribution between lower limbs for an improved quality of life and functional outcomes.

Research carried out with the help of the stabilometric platform often emphasizes the fact that weight distribution on the lower-limb level can be influenced by various factors, among which the occurrence of pathologies on the lower-limb level is one [32,33].

While specific studies might differ in their methodologies, scopes, or targeted pathologies, the consensus among researchers employing stabilometric platforms appears to highlight the significance of the manner in which lower-limb pathologies influence weight distribution. These studies collectively contribute to a deeper understanding of the relationship between pathologies and weight bearing on lower limbs, influencing postural stability and gait mechanics.

The assessment of the center-of-gravity imbalances in other studies accomplished with the help of stabilometric platforms shows that the balance parameters’ perturbations may identify degenerative lesions existing in one or both knees [34,35].

Observing the relationship between lower-limb loading disparity and sagittal deviation within the group (Table 4), a noteworthy negative correlation emerges, indicated by a correlation coefficient value of r = −0.190 and a significant threshold of *p* = 0.033. This negative correlation implies that a smaller difference in loading between the two lower limbs tends to coincide with increased barycenter deviations on the anterior–posterior plane. Such deviations are indicative of postural reactions in orthostatic positions and potential postural irregularities.

Moreover, a moderate positive association is evident between the lower-limb loading difference and lateral deviation, highlighted by a correlation coefficient value of r = 0.614 and a significant threshold value of *p* = 0.000. This positive correlation emphasizes the relationship between lower-limb loading differences and frontal barycenter deviation, indicating the subjects’ plantar pressure distribution and loading patterns within the group.

This association underscores that moderate asymmetries in loading coincide with a moderate shift in the barycenter towards the supporting leg. This alignment reflects the observed loading stereotypes among individuals within the group, shedding light on the interconnected relationship between lower-limb loading asymmetries and consequent deviations in the body’s center of gravity.

Numerous authors have analyzed the reliability of center-of-gravity testing within the support polygon to determine the influence that different pathologies may have upon the parameters related to body balance and joint stability [27,28,36,37,38,39,40].

In our study group (Figure 3), significant correlations were observed, providing insights into the relationship between various parameters related to lower limb loading and deviations across different planes. Firstly, a positive statistical correlation (r = 0.196, significance threshold *p* = 0.035) was identified between healthy-lower-limb loading and sagittal deviation. This correlation signifies that asymmetrical support, predominantly favoring the healthy lower limb, requires a more precise projection of the center of gravity within the support base in the sagittal plane.

Moreover, a substantial correlation was noted between loading differences and lateral deviation, exhibiting a strong correlation coefficient of r = 0.776 and a significance threshold of *p* = 0.000. This association emphasizes the significant deviations in the center of gravity within the frontal plane, primarily due to the contribution of the healthy lower limb. This indicates the robust functional capacity of the support limb in maintaining body stability and support during both static and dynamic positions.

These correlations point to the intricate relationship between lower-limb loading disparities and deviations across different planes. They highlight how asymmetrical loading patterns, particularly with a predominant load on the healthy limb, influence the projection of the center of gravity in different planes, contributing significantly to postural control and stability in various positions. Understanding these correlations is pivotal in comprehending the nuanced adaptations and functional adjustments that occur in response to asymmetrical loading between lower limbs, aiding in devising targeted interventions for optimizing stability and balance in individuals affected by such loading asymmetries.

The comparison between lower limb loading in the control group and the study group (Table 6, Figure 1) reveals a noteworthy and statistically significant disparity (*p* = 0.000) between the loading observed in the left and right lower limbs of the control group (mean 10.42). This disparity is notably different from the mean loading distribution between the healthy and affected lower limbs within the study group (mean 24.93), favoring the healthy lower limb. This disparity accentuates a significant asymmetry in support, particularly evident in individuals suffering from severe gonarthrosis requiring total knee arthroplasty. The increased load on the healthy lower limb points to the protective response aimed at minimizing stress on the affected knee joint, shedding light on the adaptive mechanisms adopted by individuals with severe gonarthrosis to alleviate discomfort and protect the compromised joint.

Table 7 and Figure 2 highlight a striking and statistically significant difference (*p* = 0.000) evident in the mean lateral deviation difference between the two groups. The study group notably exhibited a considerably more pronounced lateral deviation tendency, with a mean of 16.50, contrasting significantly with the lateral deviation tendency in the control group, which averaged at 3.36. This substantial difference underscores a marked contrast in lateral deviation tendencies between the two groups. The disparity in lateral deviation suggests diverse patterns in body weight distribution, postural control, or potential musculoskeletal adaptations between the two groups, reflecting distinctive characteristics or conditions within each group.

Table 8 and Figure 3 highlight a significant and statistically notable difference (*p* = 0.000) in the mean sagittal deviation between the two groups. The study group displayed a decreased deviation tendency, with a mean of 3.72, whereas the control group recorded higher mean values of 5.20. Notably, the barycenter differences observed within the support polygon regarding sagittal deviation suggest a distinctive pattern. The barycenter’s predominant shift toward the healthy lower limb prompts intensified postural rebalancing reactions from individuals, aiming to reposition the center of gravity projection within the sagittal plane closer to the central area of the support polygon. This disparity in sagittal deviation tendencies indicates potential differences in postural control strategies or adaptive mechanisms adopted by the individuals in each group, reflecting variations in weight distribution and postural adjustments between the two groups.

Several authors have extensively examined the reliability of center-of-gravity testing within the support polygon to ascertain its consistency in assessing body balance and joint stability. Their analyses have focused on understanding how diverse pathologies, including musculoskeletal disorders, neurological conditions, orthopedic injuries, and systemic diseases, influence parameters associated with balance control. These parameters encompass metrics such as center-of-gravity displacement, sway velocity, and sway amplitude. By comparing healthy individuals with those affected by specific pathologies, these studies have aimed to elucidate the alterations in balance parameters, providing crucial insights into the impacts of various conditions on body stability. Moreover, the findings have carried clinical implications, suggesting the potential use of center-of-gravity testing as a diagnostic and monitoring tool while informing tailored treatment and rehabilitation strategies to improve balance control for individuals affected by specific pathologies affecting joint stability.

One of the limitations of this research lies in the fact that, in general, most of the patients had significant bilateral damage in the knees, an aspect that represented an exclusion criterion in this study.

The advanced ages of the patients and the lack of motivation to participate in functional recovery programs, along with the association of other comorbidities that contraindicate physical effort, represented further limitations of the research.

The main direction of research is represented by the periodic evaluation of the evolution of the balance and the barycenter over a period of at least 2 years in these patients in order to monitor the degree of load and comparative request of the operated limb compared to the healthy one.

## 5. Conclusions

The use of functional testing to assess body weight distribution and center-of-gravity imbalances, in the case of gonarthrosis and other joint disorders, represents a promising direction in the research on and management of these disorders, providing essential information for functional diagnosing, elaborating, and monitoring individualized functional rehabilitation plans.

The detailed understanding of the way in which joint disorders, such as gonarthrosis, affect weight distribution and balance may provide new perspectives upon the development of tailored treatments and interventions. These data may guide the therapeutic and functional rehabilitation approaches to alleviate a patient’s functional deficit.

Non-invasive functional testing can aid in evaluating inflammatory degenerative pathologies like osteoarthritis and in developing functional rehabilitation plans.

## Figures and Tables

**Table 1:** Study and control groups.

		Mean	Median	Inter-Quartile Range
Study group 41.38% male 58.62% female	Age	61.5948	62.0000	8.00
	BMI	29.3744	28.3200	5.36
Control group 45.24% male 54.76% female	Age	61.3254	62.0000	6.00
	BMI	28.5343	28.1550	5.41

**Table 2:** Difference between left-lower-limb loading and right-lower-limb loading in the control group.

Left-Lower-Limb Loading			Right-Lower-Limb Loading			*p*
Mean		Std. Dev.	Mean		Std. Dev.	
48.28	±	6.08	51.47	±	6.08	0.004 *

* *p* < 0.05.

**Table 3:** Loading difference between the patient’s healthy and affected lower limbs within the study group.

Affected-Lower-Limb Loading			Healthy-Lower-Limb Loading			*p*
Mean		Std. Dev.	Mean		Std. Dev.	
37.44	±	4.24	62.37	±	4.33	0.000 *

* *p* < 0.05.

**Table 4:** Correlation between lower-limb loading difference with sagittal deviation and lateral deviation in the control group.

	N = 126			
	Mean		Std. Dev.	r/*p*
Load difference	10.42	±	6.80	−0.190/0.033
Sagittal deviation	5.20	±	4.24	
Load difference	10.42	±	6.80	0.614/0.000
Lateral deviation	3.36	±	1.80	

**Table 5:** Correlation between lower-limb loading differences with sagittal deviation and with lateral deviation within the study group.

	N = 116			
	Mean		Std. Dev.	r/*p*
Load difference	24.93	±	8.25	0.196/0.035
Sagittal deviation	3.72	±	1.32	
Load difference	24.93	±	8.25	0.776/0.000
Lateral deviation	16.50	±	3.73	

**Table 6:** Mean loading difference between lower limbs in the control group on frontal plane and loading difference between affected-lower-limb loading and healthy-lower-limb loading in the study group.

Left- and Right-Lower-Limb Loading Control Group (N = 126)			Affected- and Healthy-Lower-Limb Loading Study Group (N = 116)			
Mean		Std. Dev.	Mean		Std. Dev.	*p*
10.42	±	6.80	24.93	±	8.25	0.000 *

* *p* < 0.05.

**Table 7:** Comparison of mean barycenter lateral deviations between the control and the study groups.

**Lateral Deviation**	**Control Group (N = 126)**			**Study Group (N = 116)**			
	**Mean**		**Std. Dev.**	**Mean**		**Std. Dev.**	***p***
	3.36	±	1.80	16.50	±	3.73	0.000 *

* *p* < 0.05.

**Table 8:** Means of barycenter sagittal deviation in the control and study groups.

**Sagittal Deviation**	**Control Group (N = 126)**			**Study Group (N = 116)**			
	**Mean**		**Std. Dev.**	**Mean**		**Std. Dev.**	***p***
	5.20	±	4.24	3.72	±	1.32	0.000 *

* *p* < 0.05.

## Data Availability

The data presented in this study may be obtained on request from the corresponding author.
